# Preventive health behaviors among people with suicide ideation using nationwide cross-sectional data in South Korea

**DOI:** 10.1038/s41598-022-14349-w

**Published:** 2022-07-08

**Authors:** Myung Ki, Hye-Young Shim, Jiseun Lim, Minji Hwang, Jiwon Kang, Kyoung-Sae Na

**Affiliations:** 1 Department of Public Health, Graduate School, Korea University, Seoul, Republic of Korea; 2 Department of Preventive Medicine, Korea University College of Medicine, Seoul, Republic of Korea; 3 BK21FOUR R&E Center for learning Health Systems, Korea University, Seoul, Republic of Korea; 4 Department of Preventive Medicine, Eulji University School of Medicine, 77, Gyeryong-ro 771beon-gil, Jung-gu, Daejeon, 34824 Republic of Korea; 5 Department of Psychiatry, Gil Medical Center, Gachon University College of Medicine, Incheon, Republic of Korea

**Keywords:** Epidemiology, Public health

## Abstract

This study aimed to investigate the association between suicide ideation and health-related behaviors and preventive health service use behaviors. We used data from the 2017 Korea National Health and Nutrition Examination Survey (KNHANES), a nationally representative survey. The final sample included 4486 participants aged 40 years or older. Preventive health behaviors were assessed for smoking, high-risk drinking, physical activities, regular meal intake, influenza vaccination, general health examination, and cancer screening. Logistic regression was used to examine the association between suicide ideation and preventive health behaviors with a series of adjustments for covariates. In general, suicide ideation was associated with unfavorable outcomes of preventive health behaviors, except for flu vaccination. For example, the crude prevalence of suicide ideation and non-suicide ideation groups were 54.3% vs. 43.7% for flu vaccination, 23.1% vs. 41.6% for physical activity, and 24.8% vs. 18.6% for high-risk alcohol drinking. After adjustment for covariates, the associations of suicide ideation with behaviors remained significant for physical activity (OR 0.52, 95% CI 0.34–0.81) and high-risk alcohol drinking (OR 2.22, 95% CI 1.34–3.69). Suicide ideation leads to the disruption of self-management of health behaviours, especially for physical activity and high-risk alcohol drinking, independently of depressive feelings.

## Introduction

An international mental health survey conducted by the World Health Organization showed that roughly one-third of people who have had suicidal thoughts over the past 12 months develop a suicide plan, and about 15–20% of them attempt suicide^1^. While around 80–90% of people recovered after a temporary experience of suicide ideation^2^, persistent or recurrent suicide ideation may become an essential step in the suicidal process, preceding suicide attempts and completed suicide^3^. Therefore, suicide ideation has been widely used as a practical screening tool in suicide prevention^4^. Preventive health behaviors are related to suicide ideation in a manner of reinforcing each other. The relationship between both is bi-directional; for example, physical inactivity is a unique component that can be both a risk factor and a result of mental problems^5–10^. This reciprocal process shapes the self-care practice and leads to the further accumulation of adversity^11^, including suicidality development.

Suicidality is often linked to personal features of self-destructive behaviors such as a self-defeating personality, aggression, and impulsivity^12,13^. Adverse health behaviors are likely to accompany self-destructive behaviors^14^. The construction of suicidal behavior involves long-term reactions to stressful life events and emotional responses and may include subsequent changes in health behaviors.

Thus, a volume of studies, including a systematic review^15,16^, have examined the influence of health behavior on the development of suicide ideation. Despite the plausibility of such direction of associations, an alternavie approach to the association, to understand the contribution of suicide ideaton to the formation of self-destruction in health behaviors, is scarce. The only prior study that examined the association of suicide ideation as a primary interest with subsequent health service utilization implicated that suicide ideation might increase outpatient and emergency department visits regardless of depression status^17^. However, including the above study, no attention has been given to the association between suicide ideation and preventive health behaviors including health-related behaviors and preventive health service use behaviors.

Associations of suicide ideation with health-related behaviors and preventive health service use behaviors may be viewed from the perspective of mental illnesses such as depression. Studies on the association between mental health and health-related behaviors mainly focused on the youth sample. Most of these studies agree that mental health is associated with worsening of behaviors. However, only a few studies included mid-to-late adulthood population^5,18–20^ and showed inconsistent findings with varying study settings and designs. For example, the mental health status in two general populations identified using a mental health scale such as the K10 scale and CES-D was not associated with alcohol drinking^5,19^. Also, a health-plan based study showed increased exercise and fiber intake after a depressive feeling^20^. Among individuals with depressive feelings, preventive health care use such as cancer screening remained intact compared to health care management such as outpatient visits^20,21^.

Likewise, no consensus has yet been reached concerning the pattern that mental illness provokes the underuse of preventive health behaviours and variation in behaiour types. When an outcome condition was defined as a mental disorder, either a broad category such as a psychiatric disorder^22^ or specific categories such as schizophrenia^23^, the use of preventive health services decreased. In contrast, when a condition is defined as mild such as psychological distress^24^ or moderate mental illness^25^, no deterioration in the use of preventive health service was observed.

However, suicide ideation, as a situational manifestation of the mental health status and a potential intervention point of suicide death, likely influences preventive health behaviors; to date, no study examined the manifestation of preventive health behaviors, including comprehensive ranges of health-related behaviors and preventive and preventive health service use behaviors among people with suicide ideation. In the present study, we aim to investigate whether and to what degree suicide ideation is detrimental to preventive health behaviors and whether these patterns vary across behaviour types.

## Methods

### Data sources

We used data from the Korea National Health and Nutrition Examination Survey (KNHANES), an ongoing, multi-component, nationally representative survey of the non-institutionalized Korean population using a multi-stage clustered probability design. Among three components of the survey—a health interview, a health examination, and a nutrition survey—we used the data from the health interview examination executed in 2017 because this was the only year when information on suicide ideation was available among the recent wave (2016–2018) of KNHANES. Initially, 4976 subjects aged ≥ 40 years were extracted because the Korean government provides national cancer screening to individuals of this age. The final 4486 (90.2%) participants were selected after excluding 490 subjects with missing values on the questionnaire about suicide ideation. This study was approved by the Institutional Review Board (IRB) of Eulji University (approval no. EU20-045). All methods were conducted following the relevant guidelines and regulations.

### Measurements

Suicide ideation was assessed by the question, "Have you ever thought of suicide seriously over the past year?". Participants who answered "Yes" or "No" to this question were classified into a suicide ideation group or non-suicide ideation group, respectively. Preventive health service use behaviors identified as participation in influenza vaccination, general health check-ups, and cancer screening were assessed using the following questions: "Have you ever been vaccinated against influenza (seasonal flu) over the past year?"; "Have you ever undergone general examination for health over the past 2 years?"; "Have you ever been screened for cancer over the past 2 years?". The following behaviors were selected; physical activities, regular meal intake, high-risk drinking, and smoking. Physical activity was assessed by the International Physical Activity Questionnaire and was defined as "Yes" if a participant practiced moderate-intensity physical activity for more than 150 min per week, vigorous-intensity physical activity for more than 75 min per week, or equivalent combination of both activities per week (e.g., 1 min of vigorous-intensity physical activity was valued as 2 min of moderate-intensity physical activity). The following questions measured regular meal intake: "How many times have you had breakfast per week over the past year?"; "How many times have you had lunch per week over the past year?"; "How many times have you had dinner per week over the past year?". The subjects who had breakfast, lunch, and dinner five to seven times per week were categorized as having a regular meal intake. High-risk drinking was defined as drinking more than seven and five drinks almost every day for men and women, respectively. The current smoking status was categorized as nonsmoker (ex-smoker or never-smoker) and current smoker.

Covariates included demographic (gender, age, and marital status), socioeconomic (education, income, economic activity, and health insurance type), and physical and mental health-related (obesity, chronic disease status, limitation of daily activity, and depressive feeling) characteristics. Marital status was categorized as single or separated (single, separated, bereaved, and divorced) and married. Education was classified as elementary or below, middle, high, and college or above. We classified income levels into low, mid-low, mid-high, and high groups after household income was divided by the square root of the number of persons in the household. Economic activity was categorized as "Yes" or "No" depending on the participants' answer to the following question: "In your last week, have you worked more than an hour for income purposes or worked as an unpaid family worker for more than 18 h?". Everyone in Korea is expected to enroll in the national health security program, either National Health Insurance or Medical Aid. And the former is divided into the following two schemes: workplace-based health insurance scheme or community-based health insurance scheme. Because companies often provide a complementary health check-up package, workplace-based health insurance beneficiaries are advantageous concerning health service utilization. Thus, the health insurance type was classified into a workplace-based and other types of health insurance, including community-based or Medical Aid. We calculated the body mass index (BMI) as the bodyweight divided by the height squared (kg/m^2^) and classified the participants as underweight (BMI < 18.5), normal (BMI ≥ 18.5 and BMI < 25), and obese (BMI ≥ 25) groups. The chronic disease status was classified as yes if an individual had one or more doctor-diagnosed chronic diseases among hypertension, dyslipidemia, stroke, myocardial infarction, angina, pulmonary tuberculosis, diabetic mellitus, and cancer. Limitation of daily activity was categorized as "Yes" or "No" depending on the participants' answer to the question, "Do you currently have any limitation in your daily life and social activities due to health problems or physical or mental disorders?".

The depressive feeling was categorized as "Yes" if the participants answered an affirmative response to the question "Have you ever felt so sad or hopeless that you had difficulties in doing daily activities almost every day for more than 2 weeks in a row during the recent 12 months?".

### Statistical analysis

PROC SURVEY procedures were applied to reflect the KNHANES design using appropriate sampling weights to obtain accurate and representative estimates of the non-institutionalized Korean population. For descriptive analysis, a Chi-square test was performed to evaluate the difference in the characteristics and preventive health behaviors between the suicide and non-suicide ideation groups. The proportion of the utilization of preventive health services and health behaviors was calculated after adjustment for all covariates using the SAS *lsmeans* statement. The association of suicide ideation with the utilization of preventive health services and health behaviors was analyzed using logistic regression with the *proc surveylogistic* procedure considering the complex survey design. Multivariate logistic analysis was performed in four steps: in model 1, demographic factors (age and gender) were included as covariates; in model 2, socioeconomic factors such as marital status, education, income, economic activity, and health insurance type were additionally included to model 1; model 3 included the health-related factors such as obesity, chronic disease status, and limitation of daily activity in addition to model 2; model 4 finally included depressive feelings in addition to model 3. We analyzed the association with model 3 after stratification with depressive feelings and presented the results in Appendix. Participants with missing values were excluded from the multiple logistic regression models. A two-tailed p-value < 0.05 was deemed statistically significant in all analyses. All analyses were performed using SAS ver. 9.4 (SAS Institute Inc., Cary, NC, USA).

## Results

**Figure 1:**
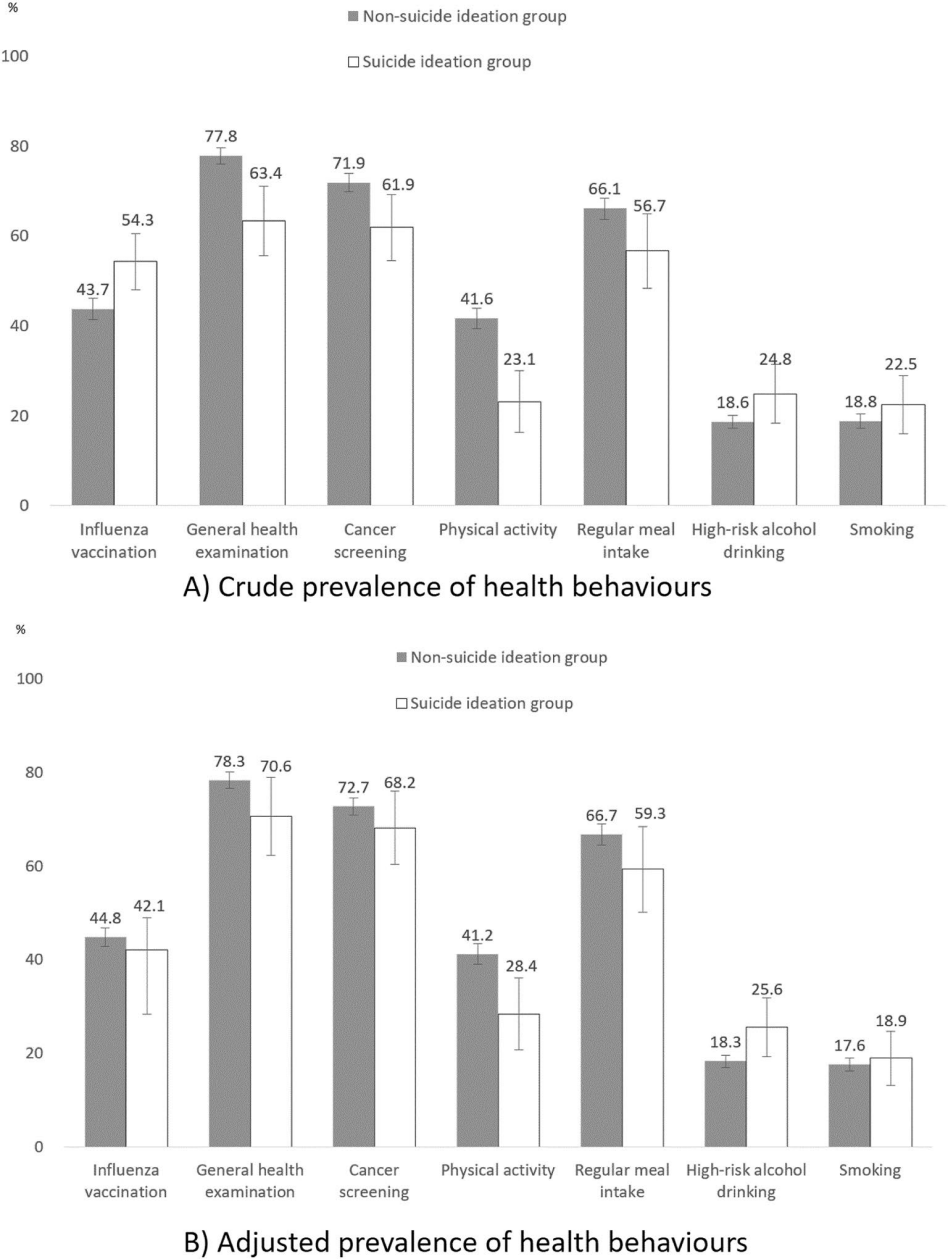
Crude and adjusted prevalence of preventive health behaviors among suicide ideation and non-suicide ideation group.

Among 4,486 participants, 244 (5.4%, weighted proportion: 5.1%) had experienced suicide ideation, and 165 out of 244 participants with suicide ideation (67.6%, weighted proportion: 68.5%) experienced depressive feelings. Compared to the non-suicide ideation group (n = 4242), the suicide ideation group (n = 244) showed a higher proportion of female, old, and single or separated people (Table 1). The suicide ideation group was vulnerable in socioeconomic status (education, income, economic activity, and health insurance type) and health-related status (chronic disease status, limitation of daily activity, and depressive feelings). The non-suicide ideation group received general health check-ups (77.8% vs. 63.4%) and cancer screening (71.9% vs. 61.9%) more and practiced physical activity (41.6% vs. 23.1%) and regular meal intake (66.1% vs. 56.7%) more and high-risk drinking (18.6% vs. 24.8%) less than suicide ideation group. On the other hand, the suicide ideation group received influenza vaccine (54.3% vs. 43.7%) more than the non-suicide ideation group, and two groups showed similar smoking prevalence (18.8% vs. 22.5%) (Fig. 1). After adjusting for all covariates, including demographic, socioeconomic, and health-related characteristics, the suicide ideation group showed more adverse health behaviors than the non-suicide ideation group regarding physical activity (28.4% vs. 41.2%) and high-risk drinking (25.6% vs. 18.3%).

**Table 1:** Characteristics and preventive health behaviors among suicide ideation group and non-suicide ideation group.

Variables		Suicide ideation group (n = 244)			Non-suicide ideation group (n = 4242)			P-value
		N	Weighted N	Weighted %	N	Weighted N	Weighted %	
Total		244	1,355,951	5.1	4242	25,485,009	94.9	
Gender	Male	95	540,433	39.9	1873	12,429,922	48.8	0.010
	Female	149	815,518	60.1	2369	13,055,087	51.2	
Age	40–49	26	195,732	14.4	1096	8,303,776	32.6	<0.001
	50–59	57	386,581	28.5	1147	7,930,877	31.1	
	60–69	70	374,934	27.7	1020	5,007,279	19.6	
	70–79	63	287,631	21.2	720	3,120,288	12.2	
	80+	28	111,073	8.2	259	1,122,789	4.4	
Marital status	Single or separated	91	448,788	35.6	759	3,895,342	16.0	<0.001
	Married	136	812,204	64.4	3324	20,437,742	84.0	
Education	Elementary or below	117	589,983	47.3	1113	5,135,649	21.3	<0.001
	Middle	33	188,706	15.1	530	3,087,189	12.8	
	High	57	348,323	28.0	1165	7,463,171	31.0	
	College or above	19	119,015	9.6	1215	8,397,628	34.9	
Income	Low	133	694,797	51.4	956	4,516,440	17.8	<0.001
	Mid-low	62	334,930	24.8	1026	5,917,509	23.3	
	Mid-high	24	127,047	9.4	1051	6,750,558	26.6	
	High	24	194,920	14.4	1197	8,188,406	32.3	
Economic activity	No	140	709,032	56.5	1643	8,682,892	36.0	<0.001
	Yes	87	545,295	43.5	2382	15,411,022	64.0	
Obesity	Underweight	10	66,021	4.9	111	644,280	2.5	0.140
	Normal	136	803,127	59.2	2586	15,525,827	61.1	
	Obese	98	486,803	35.9	1537	9,250,418	36.4	
Chronic disease status	No	75	451,971	34.6	1925	12,546,058	51.0	<0.001
	Yes	161	854,592	65.4	2192	12,063,089	49.0	
Health insurance type	Workplace-based health insurance	129	652,229	48.8	2727	16,769,947	66.6	<0.001
	Community-based health insurance or Medical aid	111	684,051	51.2	1455	8,410,873	33.4	
Limitation of daily activity	No	142	830,545	65.6	3626	22,108,716	91.6	<0.001
	Yes	87	435,031	34.4	409	2,034,839	8.4	
Depressive feelings	No	79	427,466	31.5	3837	23,228,415	91.2	<0.001
	Yes	165	928,486	68.5	402	2,247,051	8.8	
Influenza vaccination	No	87	583,416	45.7	2018	13,580,620	56.3	0.001
	Yes	143	693,682	54.3	2015	10,554,505	43.7	
General health check-ups	No	88	467,445	36.6	932	5,350,164	22.2	<0.001
	Yes	142	809,653	63.4	3099	18,774,741	77.8	
Cancer screening	No	94	486,629	38.1	1135	6,781,236	28.1	0.004
	Yes	136	790,469	61.9	2896	17,343,669	71.9	
Physical activity	No	168	961,567	76.9	2436	14,033,001	58.4	<0.001
	Yes	57	288,988	23.1	1577	10,008,803	41.6	
Regular meal intake	No	89	507,393	43.3	1151	7,488,866	33.9	0.025
	Yes	127	664,626	56.7	2614	14,606,651	66.1	
High-risk alcohol drinking	No	193	1,015,953	75.2	3560	20,743,135	81.4	0.046
	Yes	50	335,791	24.8	679	4,728,752	18.6	
Smoking	Non-smoker	195	1,051,466	77.5	3563	20,707,000	81.3	0.194
	Smoker	49	304,486	22.5	679	4,778,008	18.7

After adjustment for sex and age, the suicide ideation group still showed unfavorable practice regarding general health check-ups, cancer screening, physical activity, regular meal intake, high-risk drinking, and smoking compared with the non-suicide ideation group. In contrast, the positive association between suicide ideation and influenza vaccination disappeared. After additional adjustment for the marital status, education, income, economic activity, and type of health insurance (model 2), the significance of the negative association of suicide ideation with general health check-ups and cancer screening disappeared. The suicide ideation group still showed less physical activity and regular meal intake and more high-risk drinking and smoking than the non-suicide ideation group. In model 3, after adjusting for obesity, chronic disease status, and limitation of daily activity suicide ideation was significantly associated with less physical activity and regular meal intake and more high-risk alcohol drinking and smoking. After additional adjusting for depressive feelings, the suicide ideation group showed adverse practice regarding only physical activity (OR 0.52, 95% confidence interval 0.34–0.81) and high-risk alcohol drinking (OR 2.22, 95% confidence interval 1.34–3.69) (Table 2).

**Table 2:** Crude and adjusted associations of suicide ideation with preventive health behaviors in four steps of adjustment for covariates.

	Influenza vaccination			General health check-ups			Cancer screening			Physical activity			Regular meal intake			High-risk alcohol drinking			Smoking		
	OR	95% CI		OR	95% CI		OR	95% CI		OR	95% CI		OR	95% CI		OR	95% CI		OR	95% CI	
**Unadjusted**																					
Non-suicide ideation group	1 (reference)			1 (reference)			1 (reference)			1 (reference)			1 (reference)			1 (reference)			1 (reference)		
Suicide ideation group	1.53	1.18–1.98		0.49	0.35–0.69		0.64	0.46–0.87		0.42	0.29–0.62		0.67	0.47–0.95		1.26	0.89–1.78		1.45	1.00–2.10	
**Model 1**																					
Non-suicide ideation group	1 (reference)			1 (reference)			1 (reference)			1 (reference)			1 (reference)			1 (reference)			1 (reference)		
Suicide ideation group	0.94	0.69–1.28		0.52	0.37–0.73		0.64	0.46–0.88		0.48	0.33–0.71		0.46	0.33–0.65		2.54	1.64–3.93		2.32	1.53–3.53	
**Model 2**																					
Non-suicide ideation group	1 (reference)			1 (reference)			1 (reference)			1 (reference)			1 (reference)			1 (reference)			1 (reference)		
Suicide ideation group	0.88	0.62–1.24		0.70	0.48–1.03		0.80	0.57–1.13		0.52	0.34–0.80		0.52	0.34–0.80		2.17	1.35–3.50		1.63	1.00–2.67	
**Model 3**																					
Non-suicide ideation group	1 (reference)			1 (reference)			1 (reference)			1 (reference)			1 (reference)			1 (reference)			1 (reference)		
Suicide ideation group	0.86	0.61–1.23		0.72	0.49–1.06		0.81	0.57–1.16		0.54	0.35–0.82		0.53	0.35–0.80		2.53	1.55–4.12		1.71	1.02–2.86	
**Model 4**																					
Non-suicide ideation group	1 (reference)			1 (reference)			1 (reference)			1 (reference)			1 (reference)			1 (reference)			1 (reference)		
Suicide ideation group	0.86	0.59–1.26		0.67	0.43–1.02		0.80	0.55–1.17		0.52	0.34–0.81		0.69	0.44–1.08		2.22	1.34–3.69		1.34	0.74–2.42	

*OR* odds ratio, *CI* confidence interval.

In model 1, sex and age were adjusted.

In model 2, education, income, marital status, current economic activity, and type of health Insurance were additionally included in model 1.

In model 3, obesity, chronic diseases status, and limitation of daily activity were additionally included in model 2.

In model 4, depressive feelings were additionally included in model 3.

The association of age with preventive health care and health behaviors was various according to dependent variables. Still, a strong dose–response relationship was found in the association of age with influenza vaccination: the ORs of influenza vaccination were 1.37, 4.64, 22.92, and 24.82 among individuals in their 50 s, 60 s, 70 s, and more than 80 compared with those in their 40 s, respectively in Model 1 (Supplementary Table 1). A high-income level, workplace-based health insurance, and no chronic disease were associated with increased utilization of preventive health services and favorable health behaviors in model 4 (Supplementary Table 1).

After stratification with depressive feelings, suicide ideation was more strongly associated with adverse health behaviors among people without depressive feelings. Among them, suicide ideation was associated with unfavorable practice regarding general health check-ups, cancer screening, physical activity, high-risk drinking, and smoking after adjusting all covariates. On the other hand, in people with depressive feelings, the suicide ideation group practiced physical activity and regular meal intake less than the non-suicide ideation group after adjusting all covariates (Supplementary Table 2).

## Discussion

In this cross-sectional study using national representative data, suicide ideation showed an association with adverse health-related behaviors and underuse of preventive health services. After adjustment for all covariates, including depressive feelings, suicide ideation showed associations only with a low prevalence of physical activity (28.4% vs. 41.2%) and high-risk alcohol drinking (25.6% vs. 18.3%). To our best knowledge, this study is the first to investigate the association of suicide ideation with preventive health behaviors, including comprehensive ranges of health-related behaviors and preventive health service use behaviors.

### Comparison with previous studies

In the current study, suicide ideation was generally associated with health behaviors such as smoking, physical inactivity, high-risk drinking, and non-regular meal intake. Our results agree with previous studies reporting a positive association between mental health problems and adverse health behaviors^5,7,18–20,26^. A general explanation for the relationship between suicide ideation and health-related behaviors could be derived from psychological issues, such as withdrawal of interest, losing control of self-integrity, and tolerable risk-taking as a kind of self-harm^19^. Additionally, in the current study, the associations were particularly evident for high-risk alcohol drinking and physical inactivity even after accounting for and stratifying with depressive feelings. The strong associations of suicide ideation with these two behaviors suggest that they are strongly related to further impairment of self-management in the process of suicidality. For example, the association between mental health problems and alcohol drinking was amplified when the risk behavior was measured with high-risk drinking (e.g., everyday drinking^7^ and 5–7 drinks at a time in the current study), compared with regular alcohol use^19^. The confounding effect of depressive feelings may partly explain the lack of an association with smoking in the present study. Additionally, this may be partly explained by the possibility that elderly smokers showed low frailty and low mortality with selective survival^27^. Thus, smoking may be less useful as an indicator of adverse behavioral status in this age group, and older people comprise a large proportion of individuals with suicide ideation.

After considering covariates, there was no association between suicide ideation and preventive health service use behaviors in the current study. This finding was similar to some previous studies based on general populations^21,24^. However, it was inconsistent with other studies mainly derived from specialist mental health clinics^22,28^, which reported the underuse of preventive health services among those with mental health problems. Two explanations are possible regarding this discrepancy around studies. The self-reported mental health scale commonly used in the general population survey such as the current study is a blunt measure compared with a doctor-diagnosed disorder. Also, the general population sample did not include institutionalized patients, leading to under-representing mental patients in general population studies, unlike studies with patients of a hospital and a mental health specialist clinic. Second, no deterioration of preventive health service use behavior may be attributed to the demographic and socioeconomic characteristics related to the national health services or private screening services. This explanation is supported by the finding that the adverse impact of suicide ideation on the use of preventive health services turned insignificant after adjustment for age, sex, education, income, marital status, current economic activity, and type of health insurance. This result implies that preventive health services are maintained even when health risk behaviors are aggravated. In Korea, health-related behaviors are mainly under the individual's responsibility, while the use of preventive health services is universal under the national insurance coverage once eligibility criteria (primarily by age) are met. In addition, workplace-based health insurance beneficiaries and those of higher income earners who benefits from extra private health insurance are advantageous in receiving general health check-ups and cancer screening. This implies a relatively weaker association of suicide ideation with preventive health services use behaviors, compared to health behavior.

The stratification analysis showed an interesting finding of a more significant association between suicidal ideation and adverse health behavior in the non-depressive group. Suicidal people without depressive feelings might be more self-defeating and impulsive, leading to more negative health behaviors. Further research using more precise tools for measuring depression is necessary to understand suicidal people without depression and observe their health behaviors.

The current study is the first to explore that suicide ideation is associated with adverse health behaviors independently of other risk factors, including socioeconomic status and depressive feelings. This attempt is consistent with a line of studies^29,30^, including a meta-analysis^31^ that emphasized suicide ideation as an independent domain of mental suffering. This implies that suicide ideation is likely to be viewed in the light of the successive process toward self-destruction and an intervention to inhibit further deterioration of suicide ideation needs to address its wider context. Influences of suicide ideation may lead to the disruption of self-management and have to be extensively studied from various angles, including a range of behavioral changes.

### Methodological consideration

The present study had some methodological limitations. First, the KNHANES data are based on a cross-sectional study. Therefore, adverse health behaviors shown in the suicide ideation group may be attributed to the effect of suicide ideation on health behaviors and the influence of health behaviors on suicide ideation. However, the important point of our results is that the vulnerability of people with suicide ideation is not limited to mental states, but it can be extended to physical conditions mediated by unfavorable preventive health behaviors. This point can be drawn regardless of the direction of the association between suicide ideation and preventive health behaviors. Furthermore, the current study has first examined the pattern of preventive health behaviors related to suicide ideation concordant with other characteristics and the experience of depressive feelings.

Second, we measured depressive feelings using a single questionnaire in the present study, not using depression screening tools such as PHQ-9 or CES-D. Therefore, the validity of the measurement for depressive feelings cannot be guaranteed. Finally, all variables were measured by the recall of participants, and some degree of misclassification could occur, especially among the suicide ideation group. However, there is no expected pattern of recall about preventive health behaviors among the suicide ideation group, and this error would tend to attenuate true associations.

## Conclusion

In summary, the current study presented the patterns of association between suicide ideation and preventive health behaviors. Among individuals with suicide ideation, adverse health behaviors, in particular, physical activity and high-risk alcohol drinking, showed a closer link with suicide ideation, while preventive health service use behaviors were primarily maintained. It implicates the need for the promotion of preventive health behaviors, especially physical activity and high-risk alcohol drinking, among people with suicide ideation.

## Supplementary Information

Supplementary Tables.

## Data Availability

The datasets used and/or analysed during the current study can be accessed after logging in with an e-mail address from Korea National Health and Nutritional Examination Survey (KNHANES) website (https://knhanes.kdca.go.kr/knhanes/eng/index.do).
