# Author Correction: Preventive health behaviors among people with suicide ideation using nationwide cross-sectional data in South Korea

**DOI:** 10.1038/s41598-022-17355-0

**Published:** 2022-08-24

**Authors:** Myung Ki, Hye-Young Shim, Jiseun Lim, Minji Hwang, Jiwon Kang, Kyoung-Sae Na

**Affiliations:** 1 Department of Public Health, Graduate School, Korea University, Seoul, Republic of Korea; 2 Department of Preventive Medicine, Korea University College of Medicine, Seoul, Republic of Korea; 3 BK21FOUR R&E Center for learning Health Systems, Korea University, Seoul, Republic of Korea; 4 Department of Preventive Medicine, Eulji University School of Medicine, 77, Gyeryong-ro 771beon-gil, Jung-gu, Daejeon, 34824 Republic of Korea; 5 Department of Psychiatry, Gil Medical Center, Gachon University College of Medicine, Incheon, Republic of Korea

Correction to: *Scientific Reports* https://doi.org/10.1038/s41598-022-14349-w, published online 08 July 2022

The original version of this Article contained errors.

In Table 1, the numbers in the “P-value” column were inconsistent. The original Table 1 and accompanying legend appear below.

**Table 1:** Characteristics and preventive health behaviors among suicide ideation group and non-suicide ideation group.

Variables	Suicide ideation group (n = 244)			Non-suicide ideation group (n = 4242)			P-value
	N	Weighted N	Weighted %	N	Weighted N	Weighted %	
Total	244	1,355,951	5.1	4242	25,485,009	94.9	
**Gender**							
Male	95	540,433	39.9	1873	12,429,922	48.8	0.01
Female	149	815,518	60.1	2369	13,055,087	51.2	
**Age**							
40–49	26	195,732	14.4	1096	8,303,776	32.6	< 0.0001
50–59	57	386,581	28.5	1147	7,930,877	31.1	
60–69	70	374,934	27.7	1020	5,007,279	19.6	
70–79	63	287,631	21.2	720	3,120,288	12.2	
80+	28	111,073	8.2	259	1,122,789	4.4	
**Marital status**							
Single or separated	91	448,788	35.6	759	3,895,342	16.0	< 0.0001
Married	136	812,204	64.4	3324	20,437,742	84.0	
**Education**							
Elementary or below	117	589,983	47.3	1113	5,135,649	21.3	< 0.0001
Middle	33	188,706	15.1	530	3,087,189	12.8	
High	57	348,323	28.0	1165	7,463,171	31.0	
College or above	19	119,015	9.6	1215	8,397,628	34.9	
**Income**							
Low	133	694,797	51.4	956	4,516,440	17.8	< 0.0001
Mid-low	62	334,930	24.8	1026	5,917,509	23.3	
Mid-high	24	127,047	9.4	1051	6,750,558	26.6	
High	24	194,920	14.4	1197	8,188,406	32.3	
**Economic activity**							
No	140	709,032	56.5	1643	8,682,892	36.0	< 0.0001
Yes	87	545,295	43.5	2382	15,411,022	64.0	
**Obesity**							
Underweight	10	66,021	4.9	111	644,280	2.5	0.140
Normal	136	803,127	59.2	2586	15,525,827	61.1	
Obese	98	486,803	35.9	1537	9,250,418	36.4	
**Chronic disease status**							
No	75	451,971	34.6	1925	12,546,058	51.0	< 0.0001
Yes	161	854,592	65.4	2192	12,063,089	49.0	
**Health insurance type**							
Workplace-based health insurance	129	652,229	48.8	2727	16,769,947	66.6	< 0.0001
Community-based health insurance or Medical aid	111	684,051	51.2	1455	8,410,873	33.4	
**Limitation of daily activity**							
No	142	830,545	65.6	3626	22,108,716	91.6	< 0.0001
Yes	87	435,031	34.4	409	2,034,839	8.4	
**Depressive feelings**							
No	79	427,466	31.5	3837	23,228,415	91.2	< 0.0001
Yes	165	928,486	68.5	402	2,247,051	8.8	
**Influenza vaccination**							
No	87	583,416	45.7	2018	13,580,620	56.3	0.001
Yes	143	693,682	54.3	2015	10,554,505	43.7	
**General health check-ups**							
No	88	467,445	36.6	932	5,350,164	22.2	< 0.0001
Yes	142	809,653	63.4	3099	18,774,741	77.8	
**Cancer screening**							
No	94	486,629	38.1	1135	6,781,236	28.1	0.004
Yes	136	790,469	61.9	2896	17,343,669	71.9	
**Physical activity**							
No	168	961,567	76.9	2436	14,033,001	58.4	< 0.0001
Yes	57	288,988	23.1	1577	10,008,803	41.6	
**Regular meal intake**							
No	89	507,393	43.3	1151	7,488,866	33.9	0.025
Yes	127	664,626	56.7	2614	14,606,651	66.1	
**High-risk alcohol drinking**							
No	193	1,015,953	75.2	3560	20,743,135	81.4	0.046
Yes	50	335,791	24.8	679	4,728,752	18.6	
**Smoking**							
Non-smoker	195	1,051,466	77.5	3563	20,707,000	81.3	0.194
Smoker	49	304,486	22.5	679	4,778,008	18.7	

Additionally, in Table 2, values were missing in Model 2 for ‘Suicide ideation group’. The correct and incorrect values appear below.

Incorrect:
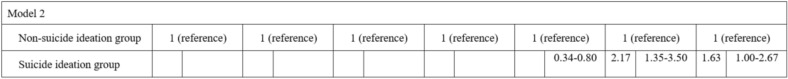


Correct:
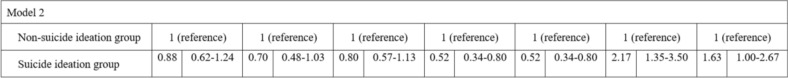


In the legend of Table 2,

“Crude and adjusted associations of suicide ideation with preventive health behaviors in three steps of adjustment for covariates.”

now reads:

“Crude and adjusted associations of suicide ideation with preventive health behaviors in four steps of adjustment for covariates.”

Furthermore, in the Methods section, under the subheading ‘Statistical analysis’,

“Multivariate logistic analysis was performed in three steps: in model 1, demographic factors (age and gender) were included as covariates; in model 2, socioeconomic factors such as marital status, education, income, economic activity, and health insurance type were additionally included to model 1; model 3 included the health-related factors such as obesity, chronic disease status, and limitation of daily activity in addition to model 2; model 4 finally included depressive feelings in addition to model 3.”

now reads:

“Multivariate logistic analysis was performed in four steps: in model 1, demographic factors (age and gender) were included as covariates; in model 2, socioeconomic factors such as marital status, education, income, economic activity, and health insurance type were additionally included to model 1; model 3 included the health-related factors such as obesity, chronic disease status, and limitation of daily activity in addition to model 2; model 4 finally included depressive feelings in addition to model 3.”

Finally, in the Results,

“After additional adjusting for depressive feelings, the suicide ideation group showed adverse practice regarding only physical activity (OR 0.52, 95% confidence interval 0.34–0.81) and high-risk alcohol drinking (OR 2.53, 95% confidence interval 1.55–4.12) (Table 2).”

now reads:

“After additional adjusting for depressive feelings, the suicide ideation group showed adverse practice regarding only physical activity (OR 0.52, 95% confidence interval 0.34–0.81) and high-risk alcohol drinking (OR 2.22, 95% confidence interval 1.34–3.69) (Table 2).”

“A high-income level, workplace-based health insurance, and no chronic disease were associated with increased utilization of preventive health services and favorable health behaviors in model 3 (Supplementary Table 1).”

now reads:

“A high-income level, workplace-based health insurance, and no chronic disease were associated with increased utilization of preventive health services and favorable health behaviors in model 4 (Supplementary Table 1).”

The original Article has been corrected.

