# Supplementary material for: Preventive health behaviors among people with suicide ideation using nationwide cross-sectional data in South Korea
Source: Sci Rep. 2022 Jul 8;12:11615. doi: 10.1038/s41598-022-14349-w (PMC9270366; doi:10.1038/s41598-022-14349-w)
Supplement: Supplementary file 1 — Supplementary Tables. [file 41598_2022_14349_MOESM1_ESM.docx]

Supplementary table 1. Associations of suicide ideation and the characteristics of participants with preventive health behaviors in three steps of adjustment for covariates

|  | Influenza vaccination | | | General health check-ups | | | Cancer screening | | | Physical activity | | | Regular meal intake | | | High-risk alcohol drinking | | | Smoking | | |
| --- | --- | --- | --- | --- | --- | --- | --- | --- | --- | --- | --- | --- | --- | --- | --- | --- | --- | --- | --- | --- | --- |
|  | OR | 95% CI | | OR | 95% CI | | OR | 95% CI | | OR | 95% CI | | OR | 95% CI | | OR | 95% CI | | OR | 95% CI | |
| ***Model 1*** |  |  |  |  |  |  |  |  |  |  |  |  |  |  |  |  |  |  |  |  |  |
| Suicide ideation |  |  |  |  |  |  |  |  |  |  |  |  |  |  |  |  |  |  |  |  |  |
| No | 1 (reference) | | | 1 (reference) | | | 1 (reference) | | | 1 (reference) | | | 1 (reference) | | | 1 (reference) | | | 1 (reference) | | |
| Yes | 0.94 | 0.69-1.28 | | 0.52 | 0.37-0.73 | | 0.64 | 0.46-0.88 | | 0.48 | 0.33-0.71 | | 0.46 | 0.33-0.65 | | 2.54 | 1.64-3.93 | | 2.32 | 1.53-3.53 | |
| Gender |  |  |  |  |  |  |  |  |  |  |  |  |  |  |  |  |  |  |  |  |  |
| Male | 1 (reference) | | | 1 (reference) | | | 1 (reference) | | | 1 (reference) | | | 1 (reference) | | | 1 (reference) | | | 1 (reference) | | |
| Female | 1.44 | 1.24-1.66 | | 0.89 | 0.76-1.05 | | 1.29 | 1.12-1.49 | | 0.89 | 0.77-1.04 | | 0.77 | 0.66-0.90 | | 0.12 | 0.09-0.16 | | 0.07 | 0.05-0.09 | |
| Age |  |  |  |  |  |  |  |  |  |  |  |  |  |  |  |  |  |  |  |  |  |
| 40-49 | 1 (reference) | | | 1 (reference) | | | 1 (reference) | | | 1 (reference) | | | 1 (reference) | | | 1 (reference) | | | 1 (reference) | | |
| 50-59 | 1.37 | 1.10-1.70 | | 1.00 | 0.79-1.27 | | 1.12 | 0.90-1.39 | | 0.90 | 0.75-1.07 | | 1.80 | 1.44-2.24 | | 0.81 | 0.65-1.03 | | 0.65 | 0.51-0.83 | |
| 60-69 | 4.64 | 3.68-5.86 | | 1.24 | 0.99-1.56 | | 1.31 | 1.06-1.61 | | 0.63 | 0.51-0.79 | | 3.97 | 3.05-5.18 | | 0.57 | 0.45-0.74 | | 0.41 | 0.31-0.54 | |
| 70-79 | 22.92 | 17.46-30.09 | | 0.68 | 0.53-0.87 | | 0.83 | 0.67-1.04 | | 0.50 | 0.40-0.63 | | 5.70 | 4.24-7.68 | | 0.23 | 0.16-0.33 | | 0.25 | 0.18-0.36 | |
| 80+ | 24.82 | 15.37-40.10 | | 0.33 | 0.23-0.47 | | 0.36 | 0.26-0.50 | | 0.27 | 0.18-0.40 | | 5.74 | 3.62-9.11 | | 0.12 | 0.06-0.24 | | 0.22 | 0.12-0.40 | |
| ***Model 2*** |  |  |  |  |  |  |  |  |  |  |  |  |  |  |  |  |  |  |  |  |  |
| Suicide ideation |  |  |  |  |  |  |  |  |  |  |  |  |  |  |  |  |  |  |  |  |  |
| No | 1 (reference) | | | 1 (reference) | | | 1 (reference) | | | 1 (reference) | | | 1 (reference) | | | 1 (reference) | | | 1 (reference) | | |
| Yes | 0.88 | 0.62-1.24 | | 0.70 | 0.48-1.03 | | 0.80 | 0.57-1.13 | | 0.52 | 0.34-0.80 | | 0.52 | 0.34-0.80 | | 2.17 | 1.35-3.50 | | 1.63 | 1.00-2.67 | |
| Gender |  |  |  |  |  |  |  |  |  |  |  |  |  |  |  |  |  |  |  |  |  |
| Male | 1 (reference) | | | 1 (reference) | | | 1 (reference) | | | 1 (reference) | | | 1 (reference) | | | 1 (reference) | | | 1 (reference) | | |
| Female | 1.28 | 1.09-1.52 | | 0.97 | 0.80-1.17 | | 1.39 | 1.17-1.65 | | 0.95 | 0.80-1.12 | | 0.79 | 0.65-0.95 | | 0.10 | 0.08-0.14 | | 0.05 | 0.04-0.07 | |
| Age |  |  |  |  |  |  |  |  |  |  |  |  |  |  |  |  |  |  |  |  |  |
| 40-49 | 1 (reference) | | | 1 (reference) | | | 1 (reference) | | | 1 (reference) | | | 1 (reference) | | | 1 (reference) | | | 1 (reference) | | |
| 50-59 | 1.29 | 1.01-1.63 | | 1.04 | 0.80-1.34 | | 1.11 | 0.88-1.41 | | 0.98 | 0.81-1.18 | | 1.80 | 1.42-2.29 | | 0.67 | 0.51-0.88 | | 0.54 | 0.40-0.72 | |
| 60-69 | 4.09 | 3.08-5.43 | | 1.65 | 1.23-2.21 | | 1.59 | 1.22-2.08 | | 0.85 | 0.68-1.08 | | 4.29 | 3.15-5.85 | | 0.43 | 0.30-0.60 | | 0.29 | 0.21-0.41 | |
| 70-79 | 18.43 | 13.03-26.07 | | 1.12 | 0.81-1.57 | | 1.27 | 0.93-1.74 | | 0.75 | 0.56-0.99 | | 6.56 | 4.61-9.32 | | 0.15 | 0.09-0.24 | | 0.18 | 0.11-0.28 | |
| 80+ | 16.86 | 9.64-29.50 | | 0.59 | 0.36-0.97 | | 0.64 | 0.41-1.02 | | 0.41 | 0.26-0.66 | | 8.11 | 4.86-13.56 | | 0.04 | 0.02-0.11 | | 0.14 | 0.07-0.26 | |
| Marital status |  |  |  |  |  |  |  |  |  |  |  |  |  |  |  |  |  |  |  |  |  |
| Single or separated | 1 (reference) | | | 1 (reference) | | | 1 (reference) | | | 1 (reference) | | | 1 (reference) | | | 1 (reference) | | | 1 (reference) | | |
| Married | 0.80 | 0.63-1.03 | | 1.44 | 1.11-1.86 | | 1.46 | 1.14-1.88 | | 0.76 | 0.61-0.95 | | 1.70 | 1.28-2.25 | | 0.97 | 0.70-1.35 | | 0.48 | 0.33-0.70 | |
| Education |  |  |  |  |  |  |  |  |  |  |  |  |  |  |  |  |  |  |  |  |  |
| Elementary or below | 1 (reference) | | | 1 (reference) | | | 1 (reference) | | | 1 (reference) | | | 1 (reference) | | | 1 (reference) | | | 1 (reference) | | |
| Middle | 0.89 | 0.67-1.19 | | 1.06 | 0.78-1.44 | | 1.01 | 0.76-1.33 | | 1.30 | 0.96-1.76 | | 1.29 | 0.95-1.75 | | 0.69 | 0.45-1.05 | | 0.69 | 0.43-1.11 | |
| High | 0.85 | 0.63-1.14 | | 1.10 | 0.84-1.45 | | 1.10 | 0.86-1.42 | | 1.60 | 1.19-2.14 | | 0.99 | 0.73-1.33 | | 0.86 | 0.60-1.24 | | 0.93 | 0.64-1.35 | |
| College or above | 0.72 | 0.52-1.00 | | 1.17 | 0.83-1.64 | | 1.00 | 0.74-1.35 | | 2.02 | 1.52-2.69 | | 1.07 | 0.76-1.51 | | 0.47 | 0.31-0.71 | | 0.47 | 0.32-0.69 | |
| Income |  |  |  |  |  |  |  |  |  |  |  |  |  |  |  |  |  |  |  |  |  |
| Low | 1 (reference) | | | 1 (reference) | | | 1 (reference) | | | 1 (reference) | | | 1 (reference) | | | 1 (reference) | | | 1 (reference) | | |
| Mid-low | 0.89 | 0.68-1.17 | | 1.28 | 0.97-1.71 | | 1.37 | 1.05-1.78 | | 1.42 | 1.12-1.80 | | 0.93 | 0.70-1.24 | | 0.88 | 0.57-1.34 | | 0.76 | 0.52-1.13 | |
| Mid-high | 1.05 | 0.79-1.40 | | 1.39 | 1.05-1.85 | | 1.57 | 1.17-2.10 | | 1.31 | 1.03-1.67 | | 0.97 | 0.70-1.35 | | 0.85 | 0.56-1.29 | | 0.87 | 0.61-1.26 | |
| High | 1.08 | 0.78-1.49 | | 1.98 | 1.43-2.76 | | 2.17 | 1.58-2.97 | | 1.40 | 1.07-1.84 | | 0.99 | 0.71-1.38 | | 1.00 | 0.65-1.55 | | 0.69 | 0.47-0.99 | |
| Economic activity |  |  |  |  |  |  |  |  |  |  |  |  |  |  |  |  |  |  |  |  |  |
| No | 1 (reference) | | | 1 (reference) | | | 1 (reference) | | | 1 (reference) | | | 1 (reference) | | | 1 (reference) | | | 1 (reference) | | |
| Yes | 0.89 | 0.74-1.07 | | 1.27 | 1.02-1.57 | | 1.13 | 0.93-1.38 | | 0.86 | 0.72-1.02 | | 0.99 | 0.81-1.22 | | 0.99 | 0.75-1.32 | | 1.33 | 0.98-1.80 | |
| Health insurance type |  |  |  |  |  |  |  |  |  |  |  |  |  |  |  |  |  |  |  |  |  |
| Local health insurance or Medicaid | 1 (reference) | | | 1 (reference) | | | 1 (reference) | | | 1 (reference) | | | 1 (reference) | | | 1 (reference) | | | 1 (reference) | | |
| Workplace health insurance | 1.20 | 0.99-1.45 | | 1.69 | 1.38-2.06 | | 1.37 | 1.12-1.67 | | 1.29 | 1.09-1.53 | | 1.28 | 1.06-1.56 | | 0.93 | 0.76-1.15 | | 0.84 | 0.66-1.07 | |
| ***Model 3*** |  |  |  |  |  |  |  |  |  |  |  |  |  |  |  |  |  |  |  |  |  |
| Suicide ideation |  |  |  |  |  |  |  |  |  |  |  |  |  |  |  |  |  |  |  |  |  |
| No | 1 (reference) | | | 1 (reference) | | | 1 (reference) | | | 1 (reference) | | | 1 (reference) | | | 1 (reference) | | | 1 (reference) | | |
| Yes | 0.86 | 0.59-1.26 | | 0.67 | 0.43-1.02 | | 0.80 | 0.55-1.17 | | 0.52 | 0.34-0.81 | | 0.69 | 0.44-1.08 | | 2.22 | 1.34-3.69 | | 1.34 | 0.74-2.42 | |
| Gender |  |  |  |  |  |  |  |  |  |  |  |  |  |  |  |  |  |  |  |  |  |
| Male | 1 (reference) | | | 1 (reference) | | | 1 (reference) | | | 1 (reference) | | | 1 (reference) | | | 1 (reference) | | | 1 (reference) | | |
| Female | 1.34 | 1.13-1.59 | | 0.96 | 0.80-1.16 | | 1.41 | 1.19-1.68 | | 0.94 | 0.79-1.12 | | 0.79 | 0.65-0.96 | | 0.10 | 0.08-0.13 | | 0.05 | 0.03-0.06 | |
| Age |  |  |  |  |  |  |  |  |  |  |  |  |  |  |  |  |  |  |  |  |  |
| 40-49 | 1 (reference) | | | 1 (reference) | | | 1 (reference) | | | 1 (reference) | | | 1 (reference) | | | 1 (reference) | | | 1 (reference) | | |
| 50-59 | 1.15 | 0.91-1.46 | | 0.98 | 0.75-1.28 | | 1.03 | 0.81-1.32 | | 0.98 | 0.81-1.19 | | 1.71 | 1.34-2.19 | | 0.65 | 0.48-0.87 | | 0.55 | 0.40-0.74 | |
| 60-69 | 3.44 | 2.60-4.55 | | 1.47 | 1.10-1.97 | | 1.38 | 1.06-1.80 | | 0.86 | 0.67-1.09 | | 3.90 | 2.85-5.34 | | 0.41 | 0.29-0.58 | | 0.30 | 0.21-0.43 | |
| 70-79 | 14.86 | 10.48-21.07 | | 0.97 | 0.68-1.39 | | 1.06 | 0.77-1.46 | | 0.77 | 0.57-1.04 | | 5.58 | 3.87-8.04 | | 0.14 | 0.08-0.24 | | 0.18 | 0.11-0.30 | |
| 80+ | 13.51 | 7.66-23.81 | | 0.51 | 0.31-0.85 | | 0.53 | 0.34-0.85 | | 0.42 | 0.26-0.68 | | 6.65 | 3.91-11.32 | | 0.04 | 0.02-0.10 | | 0.14 | 0.07-0.26 | |
| Marital status |  |  |  |  |  |  |  |  |  |  |  |  |  |  |  |  |  |  |  |  |  |
| Single or separated | 1 (reference) | | | 1 (reference) | | | 1 (reference) | | | 1 (reference) | | | 1 (reference) | | | 1 (reference) | | | 1 (reference) | | |
| Married | 0.80 | 0.62-1.03 | | 1.45 | 1.12-1.87 | | 1.48 | 1.15-1.90 | | 0.76 | 0.61-0.95 | | 1.68 | 1.25-2.25 | | 0.95 | 0.68-1.32 | | 0.48 | 0.33-0.70 | |
| Education |  |  |  |  |  |  |  |  |  |  |  |  |  |  |  |  |  |  |  |  |  |
| Elementary or below | 1 (reference) | | | 1 (reference) | | | 1 (reference) | | | 1 (reference) | | | 1 (reference) | | | 1 (reference) | | | 1 (reference) | | |
| Middle | 0.92 | 0.69-1.24 | | 1.06 | 0.78-1.43 | | 1.01 | 0.76-1.33 | | 1.30 | 0.95-1.76 | | 1.28 | 0.93-1.75 | | 0.65 | 0.42-1.01 | | 0.70 | 0.43-1.13 | |
| High | 0.89 | 0.66-1.20 | | 1.10 | 0.84-1.45 | | 1.12 | 0.87-1.45 | | 1.59 | 1.18-2.14 | | 0.97 | 0.71-1.31 | | 0.83 | 0.58-1.20 | | 0.91 | 0.62-1.31 | |
| College or above | 0.78 | 0.56-1.08 | | 1.18 | 0.84-1.67 | | 1.04 | 0.77-1.41 | | 2.02 | 1.51-2.69 | | 1.06 | 0.74-1.52 | | 0.45 | 0.30-0.68 | | 0.45 | 0.30-0.67 | |
| Income |  |  |  |  |  |  |  |  |  |  |  |  |  |  |  |  |  |  |  |  |  |
| Low | 1 (reference) | | | 1 (reference) | | | 1 (reference) | | | 1 (reference) | | | 1 (reference) | | | 1 (reference) | | | 1 (reference) | | |
| Mid-low | 0.92 | 0.70-1.20 | | 1.28 | 0.95-1.72 | | 1.37 | 1.04-1.79 | | 1.42 | 1.12-1.79 | | 0.90 | 0.67-1.21 | | 0.82 | 0.54-1.26 | | 0.75 | 0.50-1.11 | |
| Mid-high | 1.08 | 0.80-1.45 | | 1.38 | 1.04-1.84 | | 1.55 | 1.15-2.10 | | 1.30 | 1.02-1.66 | | 0.92 | 0.65-1.29 | | 0.79 | 0.52-1.20 | | 0.90 | 0.62-1.29 | |
| High | 1.09 | 0.79-1.52 | | 1.94 | 1.39-2.72 | | 2.13 | 1.55-2.93 | | 1.40 | 1.06-1.84 | | 0.92 | 0.65-1.31 | | 0.94 | 0.60-1.45 | | 0.70 | 0.48-1.02 | |
| Economic activity |  |  |  |  |  |  |  |  |  |  |  |  |  |  |  |  |  |  |  |  |  |
| No | 1 (reference) | | | 1 (reference) | | | 1 (reference) | | | 1 (reference) | | | 1 (reference) | | | 1 (reference) | | | 1 (reference) | | |
| Yes | 0.90 | 0.74-1.09 | | 1.25 | 1.01-1.56 | | 1.13 | 0.93-1.39 | | 0.84 | 0.71-1.01 | | 1.00 | 0.81-1.24 | | 0.94 | 0.71-1.25 | | 1.28 | 0.95-1.74 | |
| Health insurance type |  |  |  |  |  |  |  |  |  |  |  |  |  |  |  |  |  |  |  |  |  |
| Local health insurance or Medicaid | 1 (reference) | | | 1 (reference) | | | 1 (reference) | | | 1 (reference) | | | 1 (reference) | | | 1 (reference) | | | 1 (reference) | | |
| Workplace health insurance | 1.22 | 1.01-1.47 | | 1.72 | 1.41-2.10 | | 1.39 | 1.14-1.69 | | 1.29 | 1.09-1.54 | | 1.28 | 1.05-1.56 | | 0.95 | 0.77-1.16 | | 0.85 | 0.66-1.09 | |
| Obesity |  |  |  |  |  |  |  |  |  |  |  |  |  |  |  |  |  |  |  |  |  |
| Underweight | 1 (reference) | | | 1 (reference) | | | 1 (reference) | | | 1 (reference) | | | 1 (reference) | | | 1 (reference) | | | 1 (reference) | | |
| Normal | 0.78 | 0.46-1.33 | | 0.38 | 0.23-0.64 | | 0.41 | 0.26-0.64 | | 0.62 | 0.39-0.98 | | 0.74 | 0.42-1.28 | | 0.52 | 0.20-1.34 | | 3.19 | 1.90-5.34 | |
| Obese | 0.99 | 0.83-1.19 | | 0.84 | 0.70-1.02 | | 0.92 | 0.77-1.08 | | 0.93 | 0.80-1.09 | | 0.78 | 0.66-0.92 | | 0.95 | 0.76-1.19 | | 0.88 | 0.71-1.10 | |
| Chronic disease status |  |  |  |  |  |  |  |  |  |  |  |  |  |  |  |  |  |  |  |  |  |
| No | 1 (reference) | | | 1 (reference) | | | 1 (reference) | | | 1 (reference) | | | 1 (reference) | | | 1 (reference) | | | 1 (reference) | | |
| Yes | 1.67 | 1.40-1.99 | | 1.31 | 1.08-1.59 | | 1.45 | 1.21-1.73 | | 0.99 | 0.82-1.21 | | 1.35 | 1.10-1.65 | | 1.10 | 0.83-1.46 | | 0.86 | 0.67-1.10 | |
| Limitation of daily activity |  |  |  |  |  |  |  |  |  |  |  |  |  |  |  |  |  |  |  |  |  |
| No | 1 (reference) | | | 1 (reference) | | | 1 (reference) | | | 1 (reference) | | | 1 (reference) | | | 1 (reference) | | | 1 (reference) | | |
| Yes | 1.04 | 0.77-1.41 | | 0.89 | 0.66-1.20 | | 0.95 | 0.72-1.25 | | 0.92 | 0.68-1.23 | | 1.01 | 0.73-1.39 | | 0.50 | 0.30-0.82 | | 0.68 | 0.42-1.09 | |
| ***Model 4*** |  |  |  |  |  |  |  |  |  |  |  |  |  |  |  |  |  |  |  |  |  |
| Suicide ideation |  |  |  |  |  |  |  |  |  |  |  |  |  |  |  |  |  |  |  |  |  |
| No | 1 (reference) | | | 1 (reference) | | | 1 (reference) | | | 1 (reference) | | | 1 (reference) | | | 1 (reference) | | | 1 (reference) | | |
| Yes | 0.86 | 0.59-1.26 | | 0.67 | 0.43-1.02 | | 0.80 | 0.55-1.17 | | 0.52 | 0.34-0.81 | | 0.69 | 0.44-1.08 | | 2.22 | 1.34-3.69 | | 1.34 | 0.74-2.42 | |
| Gender |  |  |  |  |  |  |  |  |  |  |  |  |  |  |  |  |  |  |  |  |  |
| Male | 1 (reference) | | | 1 (reference) | | | 1 (reference) | | | 1 (reference) | | | 1 (reference) | | | 1 (reference) | | | 1 (reference) | | |
| Female | 1.34 | 1.13-1.59 | | 0.96 | 0.80-1.16 | | 1.41 | 1.19-1.68 | | 0.94 | 0.79-1.12 | | 0.79 | 0.65-0.96 | | 0.10 | 0.08-0.13 | | 0.05 | 0.03-0.06 | |
| Age |  |  |  |  |  |  |  |  |  |  |  |  |  |  |  |  |  |  |  |  |  |
| 40-49 | 1 (reference) | | | 1 (reference) | | | 1 (reference) | | | 1 (reference) | | | 1 (reference) | | | 1 (reference) | | | 1 (reference) | | |
| 50-59 | 1.15 | 0.91-1.46 | | 0.98 | 0.75-1.28 | | 1.03 | 0.81-1.32 | | 0.98 | 0.81-1.19 | | 1.71 | 1.34-2.19 | | 0.65 | 0.48-0.87 | | 0.55 | 0.40-0.74 | |
| 60-69 | 3.44 | 2.60-4.55 | | 1.47 | 1.10-1.97 | | 1.38 | 1.06-1.80 | | 0.86 | 0.67-1.09 | | 3.90 | 2.85-5.34 | | 0.41 | 0.29-0.58 | | 0.30 | 0.21-0.43 | |
| 70-79 | 14.86 | 10.48-21.07 | | 0.97 | 0.68-1.39 | | 1.06 | 0.77-1.46 | | 0.77 | 0.57-1.04 | | 5.58 | 3.87-8.04 | | 0.14 | 0.08-0.24 | | 0.18 | 0.11-0.30 | |
| 80+ | 13.51 | 7.66-23.81 | | 0.51 | 0.31-0.85 | | 0.53 | 0.34-0.85 | | 0.42 | 0.26-0.68 | | 6.65 | 3.91-11.32 | | 0.04 | 0.02-0.10 | | 0.14 | 0.07-0.26 | |
| Marital status |  |  |  |  |  |  |  |  |  |  |  |  |  |  |  |  |  |  |  |  |  |
| Single or separated | 1 (reference) | | | 1 (reference) | | | 1 (reference) | | | 1 (reference) | | | 1 (reference) | | | 1 (reference) | | | 1 (reference) | | |
| Married | 0.80 | 0.62-1.03 | | 1.45 | 1.12-1.87 | | 1.48 | 1.15-1.90 | | 0.76 | 0.61-0.95 | | 1.68 | 1.25-2.25 | | 0.95 | 0.68-1.32 | | 0.48 | 0.33-0.70 | |
| Education |  |  |  |  |  |  |  |  |  |  |  |  |  |  |  |  |  |  |  |  |  |
| Elementary or below | 1 (reference) | | | 1 (reference) | | | 1 (reference) | | | 1 (reference) | | | 1 (reference) | | | 1 (reference) | | | 1 (reference) | | |
| Middle | 0.92 | 0.69-1.24 | | 1.06 | 0.78-1.43 | | 1.01 | 0.76-1.33 | | 1.30 | 0.95-1.76 | | 1.28 | 0.93-1.75 | | 0.65 | 0.42-1.01 | | 0.70 | 0.43-1.13 | |
| High | 0.89 | 0.66-1.20 | | 1.10 | 0.84-1.45 | | 1.12 | 0.87-1.45 | | 1.59 | 1.18-2.14 | | 0.97 | 0.71-1.31 | | 0.83 | 0.58-1.20 | | 0.91 | 0.62-1.31 | |
| College or above | 0.78 | 0.56-1.08 | | 1.18 | 0.84-1.67 | | 1.04 | 0.77-1.41 | | 2.02 | 1.51-2.69 | | 1.06 | 0.74-1.52 | | 0.45 | 0.30-0.68 | | 0.45 | 0.30-0.67 | |
| Income |  |  |  |  |  |  |  |  |  |  |  |  |  |  |  |  |  |  |  |  |  |
| Low | 1 (reference) | | | 1 (reference) | | | 1 (reference) | | | 1 (reference) | | | 1 (reference) | | | 1 (reference) | | | 1 (reference) | | |
| Mid-low | 0.92 | 0.70-1.20 | | 1.28 | 0.95-1.72 | | 1.37 | 1.04-1.79 | | 1.42 | 1.12-1.79 | | 0.90 | 0.67-1.21 | | 0.82 | 0.54-1.26 | | 0.75 | 0.50-1.11 | |
| Mid-high | 1.08 | 0.80-1.45 | | 1.38 | 1.04-1.84 | | 1.55 | 1.15-2.10 | | 1.30 | 1.02-1.66 | | 0.92 | 0.65-1.29 | | 0.79 | 0.52-1.20 | | 0.90 | 0.62-1.29 | |
| High | 1.09 | 0.79-1.52 | | 1.94 | 1.39-2.72 | | 2.13 | 1.55-2.93 | | 1.40 | 1.06-1.84 | | 0.92 | 0.65-1.31 | | 0.94 | 0.60-1.45 | | 0.70 | 0.48-1.02 | |
| Economic activity |  |  |  |  |  |  |  |  |  |  |  |  |  |  |  |  |  |  |  |  |  |
| No | 1 (reference) | | | 1 (reference) | | | 1 (reference) | | | 1 (reference) | | | 1 (reference) | | | 1 (reference) | | | 1 (reference) | | |
| Yes | 0.90 | 0.74-1.09 | | 1.25 | 1.01-1.56 | | 1.13 | 0.93-1.39 | | 0.84 | 0.71-1.01 | | 1.00 | 0.81-1.24 | | 0.94 | 0.71-1.25 | | 1.28 | 0.95-1.74 | |
| Health insurance type |  |  |  |  |  |  |  |  |  |  |  |  |  |  |  |  |  |  |  |  |  |
| Local health insurance or Medicaid | 1 (reference) | | | 1 (reference) | | | 1 (reference) | | | 1 (reference) | | | 1 (reference) | | | 1 (reference) | | | 1 (reference) | | |
| Workplace health insurance | 1.22 | 1.01-1.47 | | 1.72 | 1.41-2.10 | | 1.39 | 1.14-1.69 | | 1.29 | 1.09-1.54 | | 1.28 | 1.05-1.56 | | 0.95 | 0.77-1.16 | | 0.85 | 0.66-1.09 | |
| Obesity |  |  |  |  |  |  |  |  |  |  |  |  |  |  |  |  |  |  |  |  |  |
| Underweight | 1 (reference) | | | 1 (reference) | | | 1 (reference) | | | 1 (reference) | | | 1 (reference) | | | 1 (reference) | | | 1 (reference) | | |
| Normal | 0.78 | 0.46-1.33 | | 0.38 | 0.23-0.64 | | 0.41 | 0.26-0.64 | | 0.62 | 0.39-0.98 | | 0.74 | 0.42-1.28 | | 0.52 | 0.20-1.34 | | 3.19 | 1.90-5.34 | |
| Obese | 0.99 | 0.83-1.19 | | 0.84 | 0.70-1.02 | | 0.92 | 0.77-1.08 | | 0.93 | 0.80-1.09 | | 0.78 | 0.66-0.92 | | 0.95 | 0.76-1.19 | | 0.88 | 0.71-1.10 | |
| Chronic disease status |  |  |  |  |  |  |  |  |  |  |  |  |  |  |  |  |  |  |  |  |  |
| No | 1 (reference) | | | 1 (reference) | | | 1 (reference) | | | 1 (reference) | | | 1 (reference) | | | 1 (reference) | | | 1 (reference) | | |
| Yes | 1.67 | 1.40-1.99 | | 1.31 | 1.08-1.59 | | 1.45 | 1.21-1.73 | | 0.99 | 0.82-1.21 | | 1.35 | 1.10-1.65 | | 1.10 | 0.83-1.46 | | 0.86 | 0.67-1.10 | |
| Limitation of daily activity |  |  |  |  |  |  |  |  |  |  |  |  |  |  |  |  |  |  |  |  |  |
| No | 1 (reference) | | | 1 (reference) | | | 1 (reference) | | | 1 (reference) | | | 1 (reference) | | | 1 (reference) | | | 1 (reference) | | |
| Yes | 1.04 | 0.77-1.41 | | 0.89 | 0.66-1.20 | | 0.95 | 0.72-1.25 | | 0.92 | 0.68-1.23 | | 1.01 | 0.73-1.39 | | 0.50 | 0.30-0.82 | | 0.68 | 0.42-1.09 | |
| Depressive feelings |  |  |  |  |  |  |  |  |  |  |  |  |  |  |  |  |  |  |  |  |  |
| No | 1 (reference) | | | 1 (reference) | | | 1 (reference) | | | 1 (reference) | | | 1 (reference) | | | 1 (reference) | | | 1 (reference) | | |
| Yes | 1.01 | 0.74-1.38 | | 1.17 | 0.84-1.62 | | 1.03 | 0.78-1.37 | | 1.06 | 0.82-1.38 | | 0.58 | 0.43-0.77 | | 1.27 | 0.88-1.84 | | 1.55 | 0.96-2.48 | |

OR, odds ratio; CI, confidence interval.

Model 1 included sex and age.

Model 2 included education, household income, marriage, current economic activity, and type of health Insurance in model 1.

Model 3 included obesity, chronic disease status, and limitation of daily activity in model 2.

Model 4 included depressive feelings in model 3.

Supplementary table 2. Crude and adjusted associations of suicide ideation with preventive health behaviors among people with and without depressive feelings

|  | People without depressive feelings | | | | People with depressive feelings | | | |
| --- | --- | --- | --- | --- | --- | --- | --- | --- |
|  | Crude OR | | Adjusted OR | | Crude OR | | Adjusted OR | |
|  | Point | 95% CI | Point | 95% CI | Point | 95% CI | Point | 95% CI |
| Influenza vaccination | 1.70 | 0.95-3.05 | 1.00 | 0.58-1.71 | 1.14 | 0.79-1.66 | 0.79 | 0.47-1.33 |
| General health check-ups | 0.35 | 0.21-0.57 | 0.47 | 0.27-0.82 | 0.70 | 0.44-1.12 | 0.92 | 0.53-1.57 |
| Cancer screening | 0.42 | 0.26-0.70 | 0.50 | 0.29-0.89 | 0.95 | 0.60-1.51 | 1.11 | 0.68-1.79 |
| Physical activity | 0.41 | 0.22-0.74 | 0.42 | 0.21-0.81 | 0.50 | 0.31-0.81 | 0.58 | 0.34-0.98 |
| Regular meal intake | 0.94 | 0.53-1.68 | 0.87 | 0.43-1.76 | 0.74 | 0.45-1.19 | 0.57 | 0.33-0.99 |
| High-risk alcohol drinking | 1.17 | 0.62-2.23 | 2.50 | 1.10-5.65 | 1.83 | 1.04-3.22 | 1.92 | 0.90-4.11 |
| Smoking | 1.48 | 0.78-2.82 | 2.55 | 1.23-5.26 | 1.16 | 0.68-1.99 | 0.83 | 0.36-1.88 |

OR, odds ratio; CI, confidence interval.

OR adjusted for sex, age, education, household income, marriage, current economic activity, type of health insurance, obesity, chronic diseases status, and limitation of daily activity.
